# Robust Metabolic Responses to Varied Carbon Sources in Natural and Laboratory Strains of *Saccharomyces cerevisiae*

**DOI:** 10.1371/journal.pone.0030053

**Published:** 2012-01-09

**Authors:** Wayne A. Van Voorhies

**Affiliations:** Auburn University, United States of America

## Abstract

Understanding factors that regulate the metabolism and growth of an organism is of fundamental biologic interest. This study compared the influence of two different carbon substrates, dextrose and galactose, on the metabolic and growth rates of the yeast *Saccharomyces cerevisiae*. Yeast metabolic and growth rates varied widely depending on the metabolic substrate supplied. The metabolic and growth rates of a yeast strain maintained under long-term laboratory conditions was compared to strain isolated from natural condition when grown on different substrates. Previous studies had determined that there are numerous genetic differences between these two strains. However, the overall metabolic and growth rates of a wild isolate of yeast was very similar to that of a strain that had been maintained under laboratory conditions for many decades. This indicates that, at in least this case, metabolism and growth appear to be well buffered against genetic differences. Metabolic rate and cell number did not co-vary in a simple linear manner. When grown in either dextrose or galactose, both strains showed a growth pattern in which the number of cells continued to increase well after the metabolic rate began a sharp decline. Previous studied have reported that O_2_ consumption in *S. cerevisiae* grown in reduced dextrose levels were elevated compared to higher levels. Low dextrose levels have been proposed to induce caloric restriction and increase life span in yeast. However, there was no evidence that reduced levels of dextrose increased metabolic rates, measured by either O_2_ consumption or CO_2_ production, in the strains used in this study.

## Introduction

Yeasts in general, and the bakers yeast *Saccharomyces cerevisiae* in particular, have been extensively used for many decades to examine a wide array of biological processes, including studies on cell cycle control, gene expression regulation and control of metabolic function [1], [2], [3]. Humans have also made widespread domestic use of yeast for thousands of years, primarily either for food baking or as a fermenting agent to produce ethanol [4], [5], [6]. The large-scale industrial use of yeast has provided an initial impetus for research studies to understand the factors controlling yeast metabolic processes [7], [8]. While yeast metabolism has been intensely studied, there remain areas of controversy on issues such as the metabolic response of yeast to reduced dextrose levels or the relative use of aerobic fermentation in varied metabolic substrates [9], [10].

Most eukaryotic cells are considered respiratory and require oxygen to metabolize dextrose. But some organisms, including *S. cerevisiae*, can metabolize dextrose via fermentation, obviating the need for oxygen [11]. In high concentrations of dextrose, *S. cerevisiae* actively represses respiratory enzyme synthesis. In these conditions dextrose is metabolized via fermentation rather than respiration, even when oxygen is abundant [11], [12], [13], [14]. The repression of respiratory enzyme synthesis by fermentation activity is referred to as the Crabtree Effect, and such aerobic glycolysis is also often characteristic of mammalian cancer cells [15]. Per unit of dextrose consumed, fermentation reduces biomass by ∼5-fold, and ATP by ∼15-fold, compared to respiration. However, fermentation proceeds at much higher flux rates, leading to higher absolute growth rates [8], [12], [16]. Furthermore, the ethanol produced as a by-product of fermentation can subsequently be utilized as a non-fermentable carbon source in respiration, thus allowing for the near complete use of all available carbon [17], [18].

As dextrose levels decline *S. cerevisiae* produces and accumulates large amounts of glycogen and trehalose which provide energy storage during starvation [19]. Once dextrose is depleted, glycogen is used as an energy source to produce respiratory and gluconeogenic enzymes in the adaptation period that precedes growth on ethanol [11]. The depletion of dextrose from the medium causes *S. cerevisiae* to undergo a transition termed the diauxic shift. During this time cell growth is transiently arrested and cell metabolism is shifted towards the use of non-fermentable carbon substrates. After the diauxic shift to respiratory metabolism, carbon substrates are catabolized via mitochondrial utilization of the tricarboxylic acid (TCA) cycle and oxidative phosphorylation [12], [17], [20]. Cells then resume a period of slow growth that can last for days, during which cell density doubles. Finally, cell cultures enter stationary phase 5–7 days after the initial inoculation [19].

While dextrose is the preferred metabolic substrate of *S. cerevisiae*, it can also grow on other sugars such as galactose, sucrose [21], [22], and a variety of non-fermentable substrates such as ethanol, glycerol and acetate [11]. The growth of *S. cerevisiae* on metabolic substrates other than dextrose induces numerous metabolic changes. For example, because dextrose requires much less energy to metabolize than other substrates, *S. cerevisiae* metabolizes galactose only in the absence of dextrose. To prevent galactose metabolism in the presence of dextrose, *S. cerevisiae* has evolved a complex regulatory network that represses genes involved in galactose metabolism. When dextrose levels exceed ∼0.25% in the medium, genes involved in galactose metabolism are completely repressed [22]. Similarly, the addition of dextrose to *S. cerevisiae* cells already growing on a non-fermentable carbon source induces a variety of changes that include large increases in rates of dextrose intake, glycolysis and protein synthesis, repression of genes encoding enzymes involved in the uptake and metabolism of alternative energy sources, stress resistance and gluconeogenesis [12].

In addition to its rich history of contributions to understanding metabolic function and regulation, *S. cerevisiae* has also been used in comparative metabolic studies between laboratory-maintained and natural isolates of yeast strains (e.g [23], [24]). While laboratory conditions typically are comprised of yeast growth in log phase in high dextrose and nutrient rich medium, these conditions may select for different metabolic capabilities than those found in natural conditions, which likely consist of brief periods of nutrient pulses followed by long periods of minimal food availability [25], [26], [27].

Previous studies have compared genotypic level differences between a *S. cerevisiae* strain isolated from a natural setting to that of a common laboratory strain [28], [29], [30]. While well over 5000 genotypic differences were observed between these two strains the phenotypic effects of these changes has not been as well characterized. Studies that have looked for phenotypic changes often have focused on examining the relationship between gene expression patterns in response to environmental or genetic changes [1], [18]. While valuable, such studies may miss the overall phenotypic effects of genetic variation in an organism. For example, the relationship between specific growth rate and the levels of enzymes directly involved in *S. cerevisiae* growth and metabolism is often complex and does not co-vary in a simple linear manner [7], [31], [32]. In the study presented here, I directly compared the metabolism, metabolic rate, and growth rate of a natural yeast strain to a common laboratory strain when grown in a variety of metabolic substrates. The metabolic phenotype of the two strains was very similar under a wide range of conditions indicating that metabolism is well buffered against genotypic changes.

## Methods

### Yeast Strains and Experimental Conditions

This study used haploid derivatives of *Saccharomyces cerevisiae* strain BY4716 or RM11-1a (also referred to as BY or RM). The strain RM11-1a was originally isolated from a California vineyard in the 1990′s [29], while BY4716 is a standard strain in widespread laboratory use [33]. Freshly streaked cells were grown on YP agar (Fisher Yeast Extract, Difco Peptone and agar) at 30°C and single colonies were transferred to 10 ml of YP in a 16mm glass test tube. The YP also contained one of two different carbon sources: 2% dextrose (Sigma Chemical) (w/v), or 3% galactose (Difco) (w/v), depending on the treatment. The 10 ml culture was placed on a rotating drum at 30°C and grown overnight. When grown in 2% glucose or 3% galactose an overnight culture reached an optical density (OD660) of 1.5–2.0 by the next morning. These cultures were then diluted 1:100 in new YP medium with the same carbon source that it was originally grown in. This diluted culture was grown until it reached an OD 660 of ∼0.1 (∼3 h for yeast grown in glucose or galactose).

### Metabolic measurements

Determination of CO_2_/O_2_ fluxes was done based on previously established methods [34]. Yeast cultures grown as described above were pipetted into sterilized metabolic “boats” which were made from modified tissue culture slides (Lab-Tek, Nalge-Nunc, Naperville, Il.) and placed in metabolic chambers. The metabolic chambers were custom manufactured from 2.5 cm diameter Pyrex glass tubing and sealed with double Viton O-rings fitted into valved brass plugs. Each chamber had a volume of 50 ml. A total of 7 chambers were used in each metabolic experiment including 1 chamber that contained only YP medium. Gas flow into each chamber was controlled using a computer controlled multiplexer (Sable Systems International, Las Vegas, NV, USA). Two ml of culture was placed in the chamber, and was then flushed with CO_2_-free air of known O_2_ content, at 150 ml/minute STPD. Each chamber was sequentially flushed ∼5 times (30 s/flush) before being sealed for the metabolic sampling interval. CO_2_ was initially removed from the air stream by running compressed air through a Pure Air (MTI, Westminster, CO) gas drier to fill a 40 L air tank to a pressure of ∼800 kPa. Removal of any additional CO_2_ was accomplished by running air from this storage tank through Drierite and Ascarite gas scrubbing columns. These columns were upstream of a mass flow controller (Sierra Instruments, Monterrey, CA, USA) used to regulate airflow into the chambers and gas analyzers. After flowing through the metabolic chamber, water was removed from the air stream with a magnesium perchlorate filter before entering a CO_2_ analyzer (either a Li-Cor 6262 or 6252 CO_2_ analyzer Lincoln, NE, USA) and a dual channel Oxzilla fuel-cell O_2_ analyzer (Sable Systems). Both CO_2_/O_2_ fluxes were recorded and the respiratory quotient (RQ) calculated to determine the rate of aerobic fermentation and metabolic substrate utilization. The CO_2_ gas analysis system was zeroed daily against CO_2_-free air, and calibrated regularly against a 989 ppm certified gas standard (Air Products, Long Beach, CA). The O_2_ analyzer was calibrated prior to each experiment against well-mixed atmospheric air scrubbed of H_2_O with a column of magnesium perchlorate. The accuracy of the system was assessed through injection of known volumes of a calibration gas standard with defined amounts of CO_2_ and N_2_ and by calculating the ratio of CO_2_/O_2_ production and consumption generated from the combustion of pure ethanol [35]. Based on these methods the CO_2_/O_2_ analyzers gave readings within 2% of the predicted values. In the presence of water CO_2_ can potentially form carbonic acid. This process occurs at a relatively slow rate in the absence of catalysts such as carbonic anhydrase, but could potentially affect measures of CO_2_ flux. To check for the possible liquid-induced attenuation of the CO_2_ signal a microinjection pump was used to inject several hundred microliters of pure CO_2_ either into liquid medium or into the air space of a metabolic chamber. The chamber was then sampled using the described methods and the amount of CO_2_ contained in the sample calculated. There was no significant effect of bubbling the CO_2_ through the liquid on the total amount of CO_2_ measured compared to that of injection into an air chamber. This indicates that the conversion of CO_2_ into carbonic acid was not occurring at a rate sufficient to affect the gas measurements. Metabolic chambers were maintained at 30°C using a custom designed temperature control chamber. Temperatures in the growth chambers and metabolic chambers were monitored with Hobo data loggers (Bourne, MA).

Oxygen and CO_2_ concentrations were analyzed on yeast samples sealed in the chambers for intervals that varied from 20 to 60 min. Data were recorded using Sable Systems DATACAN data acquisition hardware and software. Data from the CO_2_/O_2_ analyzers were corrected to compensate for the time required for the gas sample to flow from the metabolic chambers to the analyzers and was analyzed using enrichment gas analysis macros. At the end of the sampling interval the final OD was measured for each chamber to determine the final yeast cell density. The samples were also checked for bacterial contamination by direct examination at 600X with a compound microscope. The samples remained in a fixed position in the metabolic chambers during the measurements. While yeast are typically grown with shaking in flasks, I found no statistically significant difference in growth rate of yeast cells with or without shaking in the chambers over the course of the experiments (data not shown).

### Growth Assays

Yeast growth was assayed in cultures grown in 2 ml of medium contained in 35 mm diameter Petri dishes at 30°C. Liquid contained in the Petri dishes would have approximately the same surface to volume ratio as the yeast samples in the metabolic chambers. To minimize evaporation from the plates the Petri dishes were sealed in a 2 L plastic container, which had a small amount of sterile water in the bottom. Growth was assayed by recording the OD660 of 3 independent samples for each time point. Yeast density is also commonly measured using an OD of 600 nm and a set of samples was read at both OD′s to calculate the relationship between densities measured at the 2 wavelengths. There was a nearly perfect correlation between optical density measured at OD 600 and 660 (R^2^>0.99 for both strains) with the OD 600 reading for a sample of yeast around 15% less than the OD 660 reading. For the OD measurements the samples were diluted as necessary with H_2_O to ensure that the OD reading was <0.70 to maintain linearity between OD and cell number. The culture from each Petri dish was used once and then discarded. The growth rate of samples grown in Petri dishes was essentially the same as that of aliquots of the same culture simultaneously grown in the metabolic chambers.

### Metabolic Rate in Varied Dextrose

To assess the influence of dextrose levels on *S. cerevisiae* metabolic rate, 100 µl of yeast grown in 2% dextrose was added to metabolic “boats” containing YP with an initial level of 0, 0.3, 0.9, 1.5, 2 or 4% dextrose (w/v). The initial starting OD of the samples was near 0.1. The sample was then sealed in a metabolic chamber which was sampled every 20 min for a 4 h period. The metabolic rate of at least six samples were assayed at each of the dextrose concentrations. The metabolic data were standardized to a relative metabolic rate of 1.0 against the second metabolic reading to control for differences in starting yeast density. The second metabolic reading was used because by this reading the gas level in the sample was in steady state with the initial gas concentration used to fill the chamber. A per cell metabolic rate was calculated for the two yeast strains by determining the number of yeast in the sample boat immediately after recording the last metabolic reading. The last metabolic reading was divided by the estimated cell number to calculate the CO_2_/O_2_ metabolic rates per cell.

### Statistics

Simple statistics were calculated using the statistical analysis function in Excel. An ANOVA analysis (Systat 5) with a Fisher LSD post-hoc test was used to determine if different dextrose levels had a significant effect on yeast metabolic rate within the 2 yeast strains. The effect of varied dextrose levels on the metabolic rate between strain BY4716 and RM11 were compared to each other using a Student's T-test corrected for multiple comparisons using a Bonferroni correction.

## Results

### Growth and metabolic rate on varied metabolic substrates

**Figure 1:**
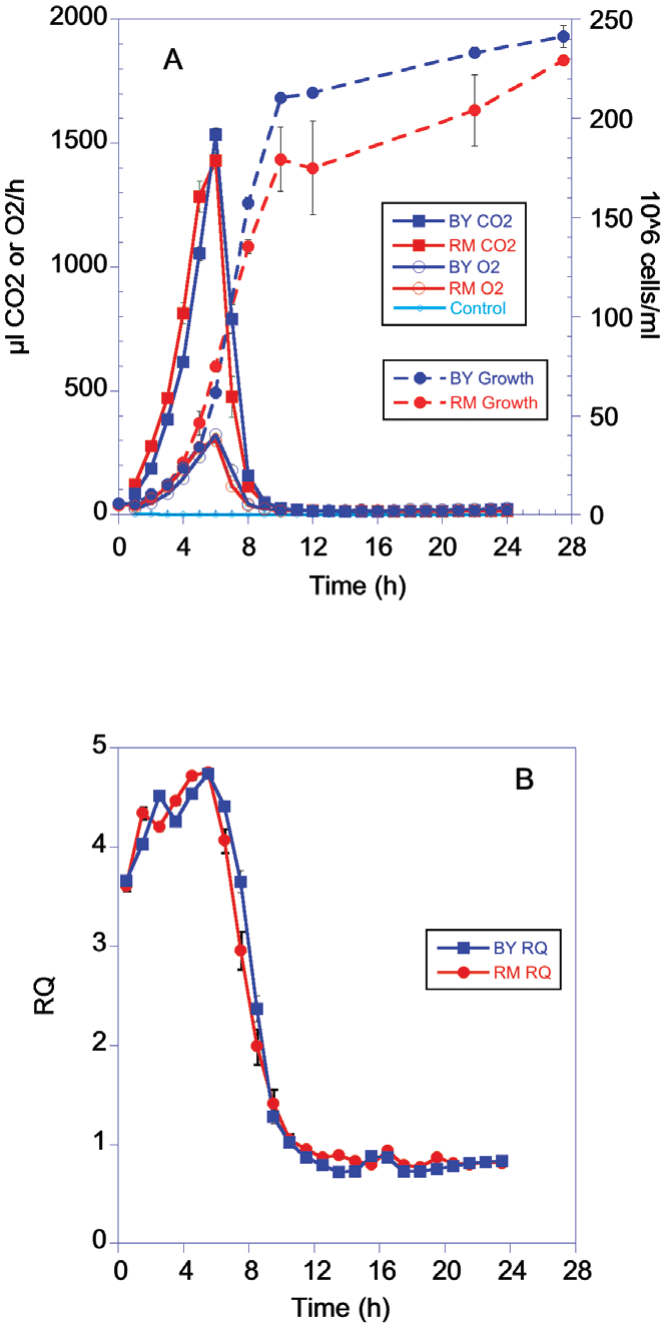
Metabolic rate, growth and respiratory quotient (RQ) of BY and RM grown in YP with 2% dextrose. Graphs are plotted using the means and sem of 6 groups for the CO_2_/O_2_ measurements and for 3 groups for the growth measurements. Cell number per ml for figures 1 and 2 were calculated based on converting an optical density reading to an estimated cell number.

As seen in figure 1A, when grown in 2% dextrose both the BY and RM strains show an exponential increase in CO_2_/O_2_ fluxes for ∼5 h followed by a sharp decline in metabolic rate. Growth rate also increased exponentially for ∼9 h and then slowed and reached a final cell density several-hundred fold higher than the starting density. The respiratory quotient (Figure 1B) increased over the first several hours and then declined sharply. This decrease in RQ is consistent with cells switching from aerobic fermentation of dextrose to the use of ethanol, or stored glycogen, as the metabolic substrates for respiration [19], [36]. The lower RQ observed during the first few hours is consistent with an increased use of respiratory metabolism during low growth rates [7], [8], [31]. The patterns of metabolic response and growth rate in dextrose were very similar between the BY laboratory strain and the RM strain isolated from a vineyard.

**Figure 2:**
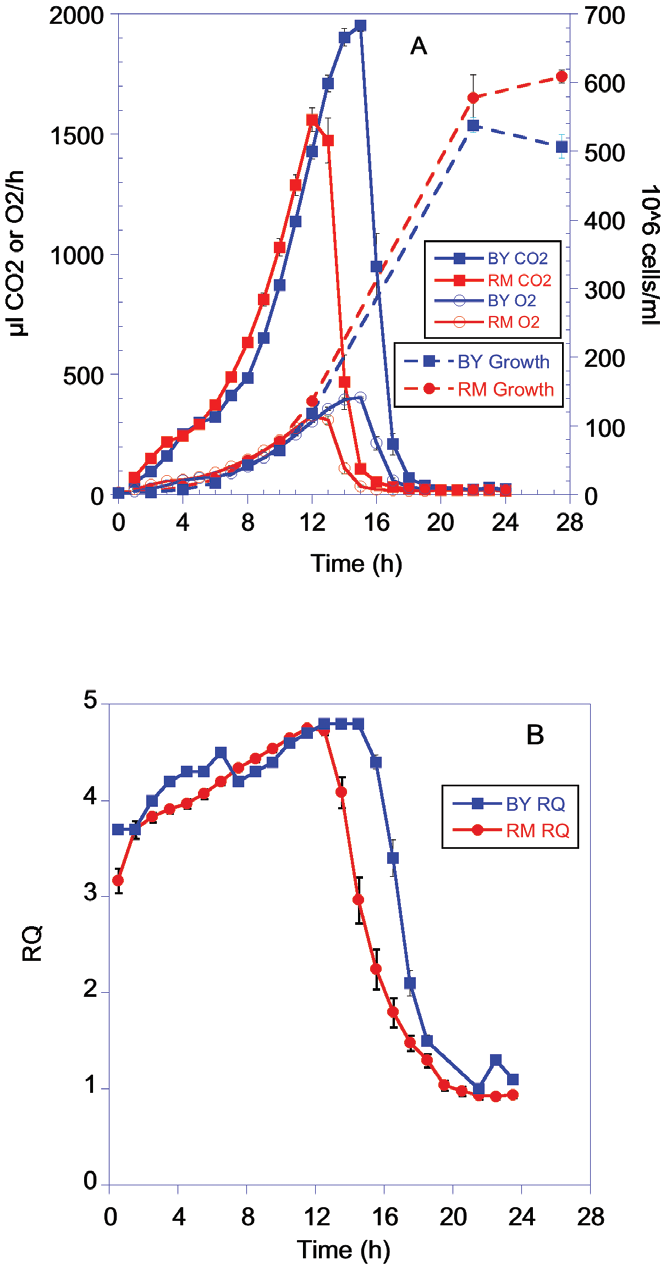
Metabolic rate, growth and RQ of BY and RM grown in YP with 3% galactose. Graphs are plotted using the means and sem of 6 groups for the CO_2_/O_2_ measurements and for 3 groups for the growth measurements.

The pattern of metabolic response for BY and RM grown in 3% galactose was similar to that on 2% dextrose (Figure 2), although there are notable differences. Compared to growth in 2% dextrose cells grown in galactose exhibited a longer lag time before metabolic and growth rates began to increase exponentially. This lag period was ∼4–6 h for growth in 3% galactose compared to ∼1 h in 2% dextrose. The final cell density obtained in galactose was also 2–3 times higher than in 2% dextrose, consistent with there being a greater biomass resource of carbon. As seen from the RQ values (Figure 2B) both strains used galactose for aerobic fermentation in a manner very similar to that seen with dextrose.

### Growth

Metabolic rate and cell number did not co-vary in a simple linear manner (Figures 1A and 2A). When grown in either 2% dextrose or 3% galactose, both strains showed a growth pattern in which the number of cells continued to increase well after the metabolic rate began a sharp decline (Figure 1A and 2A).

The highest maximum specific growth rates per hour (also called the growth rate constant) were in dextrose (µ = 0.60 for BY and RM), slightly lower in galactose (µ = 0.47 for BY, and 0.40 for RM). The difference in maximum specific growth rates of the two strains for growth in galactose was not significant (Student t-test, maximum µ, BY = 0.480±0.059; RM = 0.402±0.018. P = 0.28, df = 4). The growth rates of the two strains in galactose was also compared during 7 time points during the first 24 hours of active growth and were not significantly different (Paired t-test, mean µ, BY = 0.303±0.059*;* RM = 0.292±0.018. P = 0.60, df = 6). The growth rates on dextrose and galactose reported here are consistent with the growth rates expected on these different carbon sources [7], [9], [31], [37], [38].

### The effect of dextrose levels on metabolic rate

**Figure 3:**
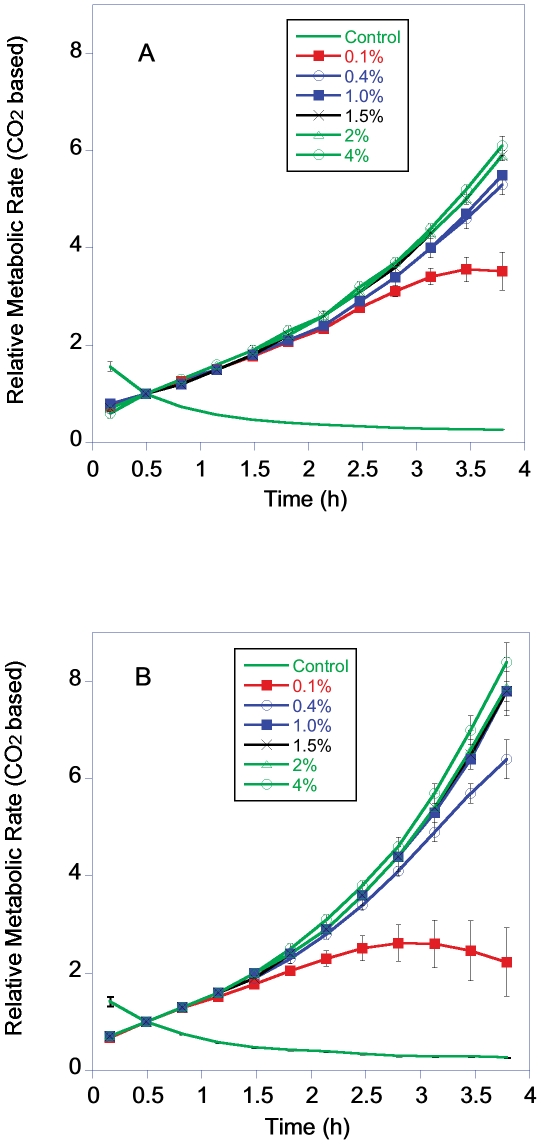
Relative metabolic rate of BY (3A) and RM (3B) grown in YP with a starting concentration of 0.1 to 4% dextrose based on CO_2_ production. Figures 3 –6 are plotted using the means and sem of 6 groups. The metabolic rate data were normalized to a value of 1.0 at the 2^nd^ metabolic reading.

**Figure 4:**
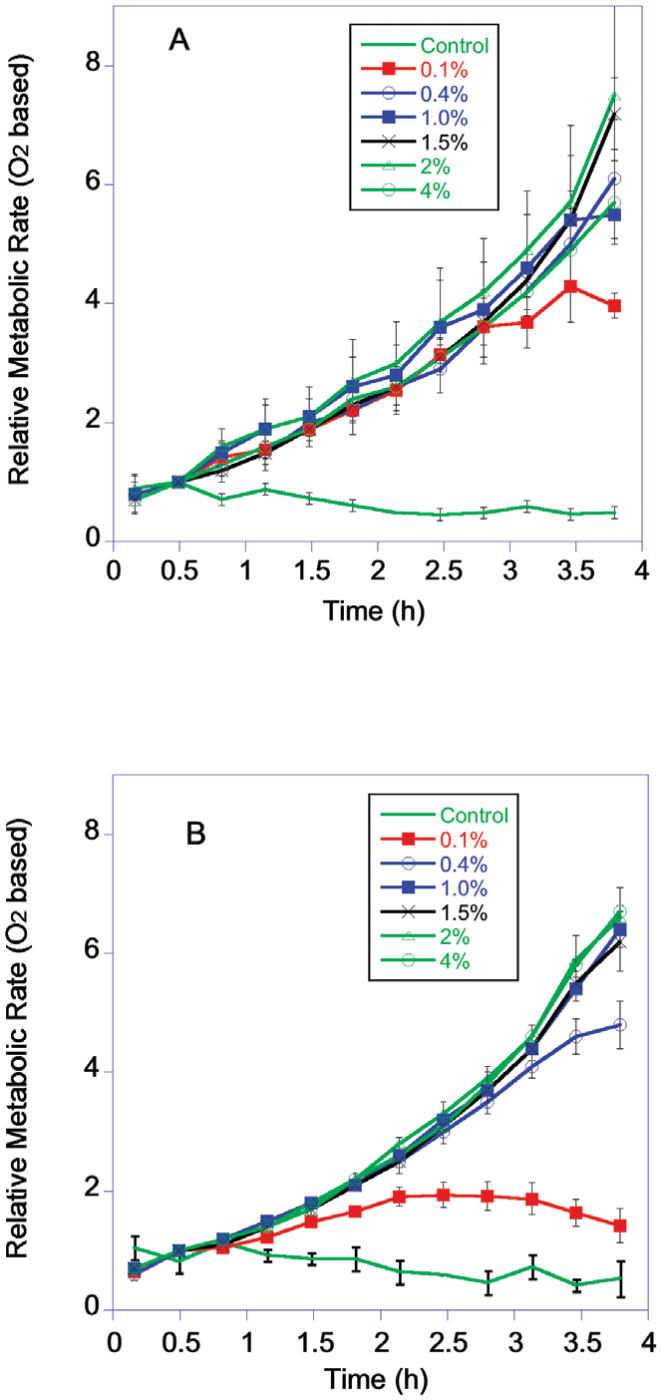
Relative metabolic rate of BY (4A) and RM (4B) grown in YP with a starting concentration of 0.1 to 4% dextrose based on O_2_ consumption.

Yeast metabolic rate, measured both as CO_2_ production (Figure 3) and O_2_ consumption (Figure 4) increased exponentially over the 4 h time course in the 5 higher dextrose levels. The yeast grown in 0.1% dextrose had an initial increase in metabolic rate similar to the groups in higher dextrose. However, the exponential increase in metabolic rate of this group ended after 1–2 h, probably due to the depletion of the available dextrose. The pattern of increase in metabolic rate was very similar between BY and RM strains (Figure 3 and 4).

**Figure 5:**
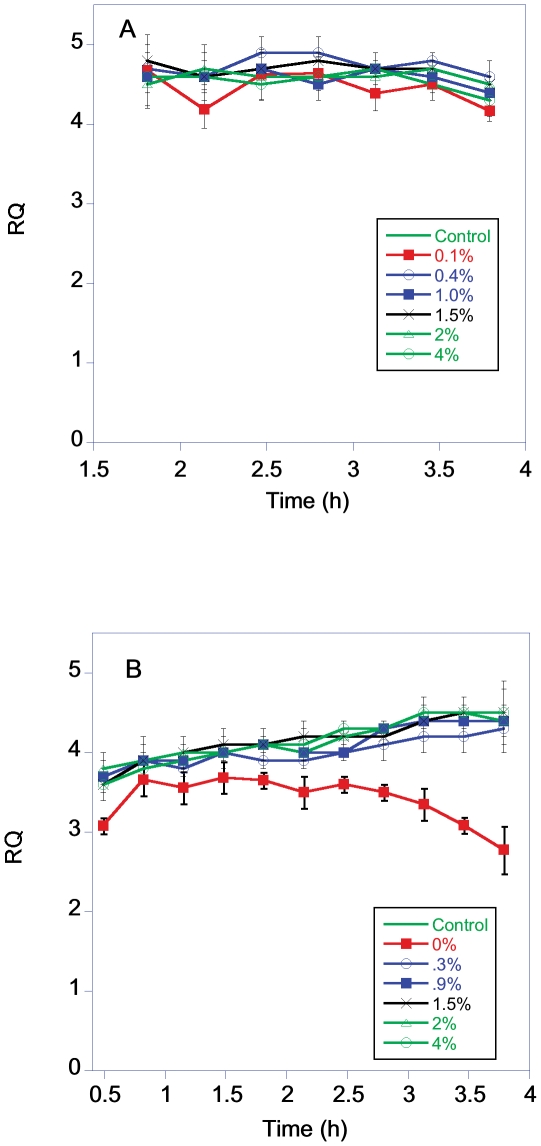
RQ of BY (4A) and RM (4B) grown in YP with a starting concentration of 0.1 to 4% dextrose.

The starting respiratory quotient of BY and RM in the varied dextrose levels was >4, consistent with the use of aerobic fermentation as the primary means of metabolism (Figure 5). The RQ of both strains remained relatively constant over the sample period. There were no significant differences in RQ over time (repeat measures ANOVA, F = 0.211, P = 0.95, df = 5), if the RQ values for the RM strain growing in 0.1% dextrose are excluded from the analysis. When this group is included there is a significant reduction in RQ starting at the 2.5 h sample interval. The RQ of BY was ∼15% higher than RM11, even if the lowest RM dextrose group is excluded (average RQ BY = 4.70±0.05, RM  = 4.05±0.03 P<<0.001 df = 563).

**Figure 6:**
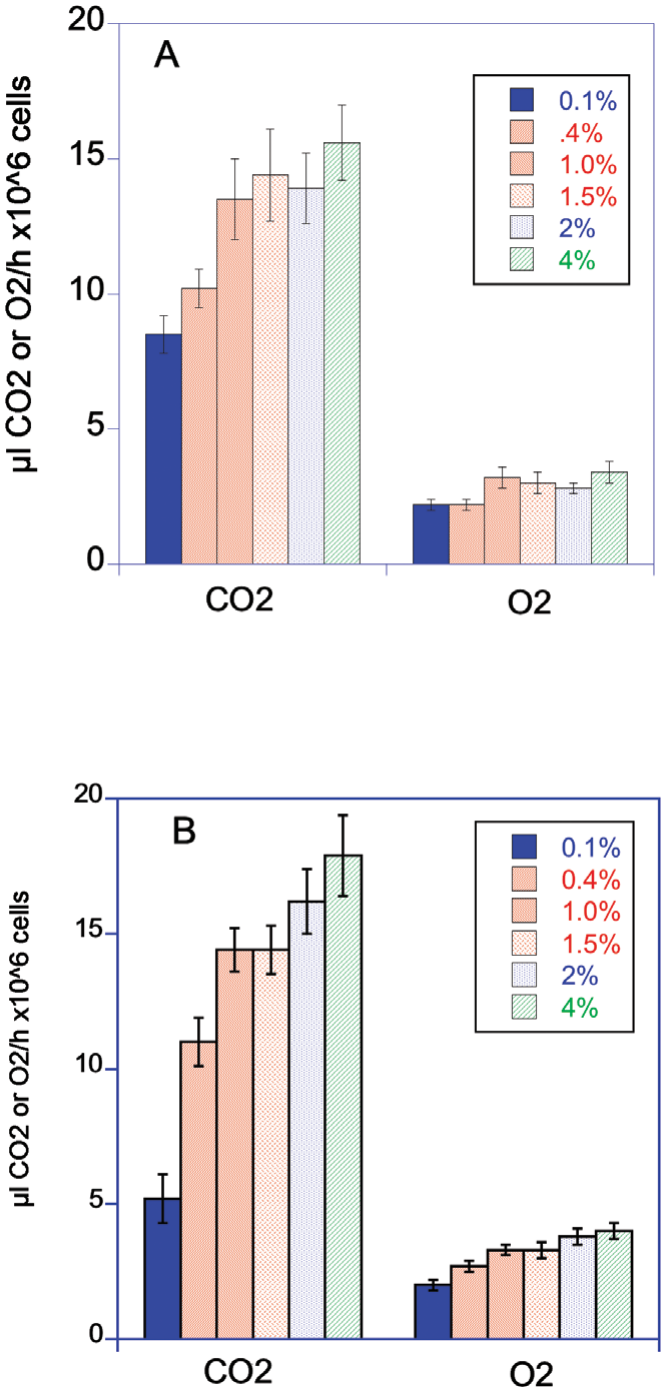
Cell specific metabolic rates of BY (4A) and RM (4B) grown in YP with a starting concentration of 0.1 to 4% dextrose. The cell specific metabolic rates are based on dividing the final metabolic rate measurement of yeast grown in different dextrose concentrations by the final number of cells in the sample chamber.

There was no indication of reduced dextrose levels increasing cellular metabolic rates as measured by rates of CO_2_/O_2_ flux (Figure 6). The metabolic rate of 0.1% dextrose groups had the lowest cell specific metabolic rate. One limitation of this result is this group had probably already started reducing its metabolic rate in response to low dextrose levels. The cell specific rate of CO_2_ production rates for the BY cells in 0.1% dextrose were significantly lower than cells in 1 to 4% dextrose and the O_2_ consumption was reduced compared to cells growing in 1.5 and 4% dextrose (CO_2_ ANOVA, F = 4.6, P = 0.003, df = 5; O_2_ ANOVA, F = 2.3, P = 0.07, df = 5 Post Hoc comparison FLSD). The cell specific rate of CO_2_ production rates for the RM cells in 0.1% dextrose were significantly lower than cells at all the other dextrose levels and the O_2_ consumption was reduced compared to cells growing in 1.0 to 4% dextrose (CO_2_ ANOVA, F = 18.3, P<0.001, df = 5; O_2_ ANOVA, F = 7.4, P = <0.001, df = 5 Post Hoc comparison FLSD). On a per cell basis the metabolic rates of RM and BY were not significantly different in the different dextrose concentrations when compared either by CO_2_ production or O_2_ consumption (Student's T-test, P = 0.01 to control for multiple comparisons).

## Discussion

### Metabolic Rates

The metabolic and growth rates of *S. cerevisiae* strains BY4716 and RM11-1a grown on dextrose and galactose varied depending on the metabolic substrate. While the metabolic substrate had a large effect on metabolism, the responses of the two strains to the different substrates were very similar. The absolute metabolic rates of the two strains were also very similar and are consistent with other values in the literature for *S. cerevisiae* [11].

The maximum observed RQ values were between 4–5 for yeast growth in dextrose and galactose similar to previously reported values [39]. An RQ of 1.0 would be expected if these sugars were being metabolized solely in aerobic respiration [36]. There have been conflicting conclusions regarding the degree to which *S. cerevisiae* normally undergoes aerobic respiration in dextrose [9]. Based on the RQ measurements observed in this study it is apparent that the yeast were extensively using aerobic fermentation to metabolize dextrose. While elevated above 1.0, the fact that the RQ was not higher than 5 indicates that respiration was not entirely suppressed in these cells. Because dextrose levels of >0.25% inhibit the expression of genes involved in respiration, it might be expected that the RQ of yeast in dextrose would be higher than during growth in galactose. This suppression of genes involved in aerobic respiration would be expected to shift metabolism towards glycosis-based metabolism which does not consume oxygen. Data from this study, however, do not support that conclusion, although a possible explanation for the lower than expected RQ values is that oxygen was being consumed by non-respiratory processes such as biosynthesis.

### Correlations between growth and metabolic rate

Growth rate, cell number and metabolic rate co-varied in a complex manner in all of the substrates. The peak in metabolic rate occurred well before the peak in growth rate for BY and RM grown in either dextrose or galactose.

A reason for the observed pattern between metabolic rate and cell number may be related to the ability of many microorganisms to anticipate changes in environmental stimuli or conditions by adapting to their temporal order of appearance [3]. It has been proposed that when *S. cerevisiae* senses a depletion in dextrose levels the cells begin to shut down metabolic processes and proceed through one final cell division [40]. It has also been observed that *S. cerevisiae* can both sense their instantaneous growth rate and anticipate that dextrose will become depleted [19], [41]. Yeast can respond to a decrease in dextrose levels by increasing the number of unbudded cells [19]. The results from this study support the hypothesis that *S. cerevisiae* anticipates the depletion of either dextrose or galactose and responds by greatly reducing metabolic rate before producing a final round of daughter cells.

The maximum specific growth rate observed in this study in dextrose was ∼0.60. This is similar to other values reported for *S. cerevisiae* that typically range from 0.42 to 0.55 [7], [9], [31], [37], [38], although my values for the maximum specific growth rate in galactose are somewhat higher than earlier reports [37].

### Metabolic rates in varied dextrose concentrations

It had previously been reported that O_2_ consumption in *S. cerevisiae* grown in medium containing 0.5% dextrose, a level proposed to induce caloric restriction, was elevated compared to yeast grown in 2% dextrose [10]. Based on this result it was hypothesized that this increase in respiration could be responsible for the increased lifespan of yeast grown in 0.5% dextrose [10]. In contrast to earlier reports my metabolic measurements, measured as CO_2_ production or O_2_ consumption, found no indication of an increase in metabolic rate in reduced levels of dextrose in either the BY or RM. A unique aspect of this study was that it measured both CO_2_ and O_2_ gas fluxes in yeast grown in varied dextrose levels. These measurements showed that reduced dextrose levels did not increase either aerobic or fermentative based metabolism in yeast.

Other studies also indicate that growth in medium containing 0.5% dextrose should not induce metabolic changes in *S. cerevisiae*. Brettel et al. reported that the metabolic and growth rate of *S. cerevisiae* remained almost constant in dextrose levels between 0.16 to 1.6% [42]. *S. cerevisiae* does switch to respiratory metabolism when the medium is depleted of dextrose, but it appears that the cells must actually be starved of dextrose before they undergo a diauxic shift [12]. This makes it unlikely that a dextrose level of 0.5% will increase rates of oxidative respiration. The fact that the RQ values remained elevated near 4 for the cultures grown in the different dextrose levels indicates that the cells were continuing to carry out aerobic fermentation at dextrose levels well below 0.5%. The one exception to this was the cells that started growth in the 0.1% dextrose. This group showed a decrease in RQ, as would be expected if the low dextrose levels were inducing a switch to mixed respiro-fermentative metabolism [31]. However the absolute metabolic rate of these cells was reduced compared to groups growing in higher dextrose levels.

While gene expression patterns in *S. cerevisiae* are very responsive to dextrose levels, the phenotypic effects of varied dextrose levels on growth and metabolism are well buffered [18]. The growth of *S. cerevisiae* is relatively insensitive to dextrose levels and remains relatively constant when dextrose levels are >0.01% [12], [43].

### Metabolism and Growth in Laboratory and Natural Strains

Although the recent evolutionary history of the BY and RM strains were likely quite different, both strains had very similar patterns of metabolism and growth. In a series of studies Kruglyak and colleagues used these same 2 strains of yeast to assay for genotypic differences between the strains [28], [29], [30]. Nearly 6000 genotypic differences were found between the strains. Despite a large number of genotypic differences, this study shows that both the metabolic and growth characteristics of the two strains are very similar. Because of their fundamental importance of metabolism and growth to the survivorship and fitness of an organism it is perhaps not surprising that the two strains showed such similar patterns.

Other studies that have compared biological variation between different strains of *S. cerevisiae* have found some traits remain relatively invariant, with others showed a wide degree of variation. Van Dijken et al. compared growth rates, biomass yields, sporulation, mating efficiency, transformation efficiency, and growth rate at which yeast began respiro-fermentation in chemostats of 4 commonly used laboratory strains [44]. They found considerable variation between the strains in all of these traits except for biomass accumulation per gram of dextrose consumed. A study comparing the metabolism and physiology in natural yeast isolates found that two sister species differed at the genotypic level but were essentially identical phenotypically in terms of growth and metabolic response to different substrates [6].

The varied metabolic capabilities of yeast make them a valuable model organism for studying factors affecting metabolism. The overall metabolic and growth rates of a wild isolate of yeast was very similar to that of a strain that had been maintained under laboratory conditions for many decades indicating that metabolism and growth appears to be well buffered against genetic differences.
